# Is It All about Surface Topography? An Intra-Individual Clinical Outcome Analysis of Two Different Implant Surfaces in Breast Reconstruction

**DOI:** 10.3390/jcm12041315

**Published:** 2023-02-07

**Authors:** Ines Schoberleitner, Angela Augustin, Daniel Egle, Christine Brunner, Birgit Amort, Bettina Zelger, Andrea Brunner, Dolores Wolfram

**Affiliations:** 1 Department of Plastic, Reconstructive and Aesthetic Surgery, Medical University of Innsbruck, Anichstraße 35, A-6020 Innsbruck, Austria; 2 Department of Obstetrics and Gynecology, Medical University of Innsbruck, Anichstraße 35, A-6020 Innsbruck, Austria; 3 Department of Radiology, Medical University of Innsbruck, Anichstraße 35, A-6020 Innsbruck, Austria; 4 Institute of Pathology, Neuropathology and Molecular Pathology, Medical University of Innsbruck, Müllerstraße 44, A-6020 Innsbruck, Austria

**Keywords:** nipple-sparing mastectomy, prophylactic implant-based breast reconstruction, SMI (silicone mammary implants), SMI surface topography, surface roughness, fibrosis, capsular contracture, aesthetic outcome, intra-individual comparison, titanized mesh implant pocket

## Abstract

The most common long-term complication of silicone breast implants (SMI) remains capsular fibrosis. The etiology of this exaggerated implant encapsulation is multifactorial but primarily induced by the host response towards the foreign material silicone. Identified risk factors include specific implant topographies. Of note, breast implant-associated anaplastic large cell lymphoma (BIA-ALCL) has only been observed in response to textured surface implants. We hypothesize that reduction of SMI surface roughness causes less host response and, hence, better cosmetic outcomes with fewer complications for the patient. A total of 7 patients received the routinely used CPX^®^4 breast expander (~60 µM Ra) and the novel SmoothSilk^®^ (~4 µM Ra), fixed prepectoral with a titanized mesh pocket and randomized to the left or right breast after bilateral prophylactic NSME (nipple-sparing mastectomy). We aimed to compare the postoperative outcome regarding capsule thickness, seroma formation, rippling, implant dislocation as well as comfortability and practicability. Our analysis shows that surface roughness is an influential parameter in controlling fibrotic implant encapsulation. Compared intra-individually for the first time in patients, our data confirm an improved biocompatibility with minor capsule formation around SmoothSilk^®^ implants with an average shell roughness of 4 µM and in addition an amplification of host response by titanized implant pockets.

## 1. Introduction

Breast augmentation with silicone mammary implants (SMI) is one of the most performed procedures in aesthetic surgery. According to the American Society of Plastic Surgeons (ASPS), officially, almost 200,000 breast augmentations were performed in the United States in 2020 [1] and current conservative estimates suggest that more than 35 million women worldwide have textured breast implants [2]. Since breast cancer is the most common cancer in females worldwide [3] and due to increasing numbers of women diagnosed with and surviving breast cancer, it is critical to stay acquainted with breast reconstructive trends. Despite significant advances in screening, diagnostic, and cancer therapies the total mastectomy numbers remain high [4]. Breast reconstruction after mastectomy significantly impacts the long-term body image, quality of life, and psychological health of affected women [5,6]. Despite clear evidence of superior long-term satisfaction after autologous breast reconstruction, the US and Europe have experienced a clear shift towards implant-based breast reconstruction. As of 2002, implants surpassed autologous techniques as the most common method of breast reconstruction in the US [7]. The benefits of implant-based reconstruction are its ease of technique, faster recovery, and avoidance of donor-site morbidity, but autologous techniques have proven to be superior in terms of long-term quality of life and aesthetic outcome [8,9,10,11].

Therefore, the motivation is high to continuously refine the technique of implant-based breast reconstruction to meet high aesthetic demands. Immediate two-stage expander (inflatable SMI)-based breast reconstruction is, according to the American Society of Plastic Surgeons (ASPS), still the most commonly chosen technique [12]. With this technique a partially filled tissue expander is placed first and, in a second stage after several months and completion of pocket formation, an exchange for a permanent implant is conducted. This approach allows to offload of initial pressure on the potentially compromised mastectomy skin flap and it allows the selection of the ideal permanent implant following an expansion period as well as to correct the implant pocket during the second stage [13,14,15]. 

Silicone is the implant material (i.e., foreign body) most widely used in routine medical practice. However, local and systemic complications in SMI carriers are still frequently reported and are controversially discussed in the literature [16,17,18,19,20,21,22,23,24,25,26,27,28,29]. The most common complication of SMI is fibrotic-implant encapsulation and peri-SMI capsular contracture, with a reported incidence from 0.5% to 50% [26,27,29,30]. The resulting clinical symptoms are pain, local tissue damage, and an unpleasing aesthetic outcome. This high variability of reported capsular contracture rates depends on many factors: different periods, types of implants used, implant locations, and others such as “defensive reporting” [17,19,20,21].

After device implantation, mechanisms of foreign body response (FBR) induce fibrosis, an excessive formation of collagenous and non-collagenous extracellular matrix (ECM) components in organs and tissues, as a reparative or reactive process [31], mainly due to the proliferation and activation of fibroblasts and myofibroblasts. There is a considerable amount of evidence highlighting fibrosis as a result of complex and sequential chronic inflammatory reactions induced by various stimuli (e.g., persistent infections, autoimmune reactions, allergic responses, chemical insults, radiation, and tissue injury) [32,33,34,35,36,37,38,39,40]. 

According to the International Organization for Standardization (ISO), 14607:2018 implants can be discriminated by a classification system based on surface topography according to surface roughness (Ra) determination by scanning electron microscopy (SEM) [41]. According to this classification, SMI surfaces are designated as smooth (Ra < 10 μm), microtextured (10 μm ≤ Ra ≤ 50 μm), or macrotextured (Ra > 50μm). Clinical studies suggest that these different surface architectures induce different foreign body immune responses and fibrosis [2,42,43,44,45,46,47,48,49,50]. Higher surface texture complexity leads to increased capsule disorganization, tissue ingrowth, and adherence; textured implants thus show a lower risk of malposition or rotation [48,51,52]. Importantly, macrotextured prostheses showed a higher risk of biofilm formation and they have been associated with the occurrence of anaplastic large cell lymphoma (BIA-ALCL) [53,54,55,56]. Due to the BIA-ALCL topic, the impact of surface roughness has gained immense attention and sales numbers of breast implants in the US show a clear shift toward smoother devices. Therefore, currently, microtextured tissue expanders are widely used, but also within this group surface roughness may vary widely. Zhang et al. showed an increase in collagen formation and fibroblast formation but a decreasing expression of inflammation cytokines with higher surface roughness in the capsule surrounding the implant in a rat model [57]. In a previous approach, we investigated the immune response of human peripheral blood mononuclear cells (PBMC) to commercially available silicone surfaces (breast implant surfaces) in vitro and could demonstrate that a decrease in surface topography reduces adherent immune cells and fibrosis-associated cytokine gene expression [46]. Correspondingly, recent in vivo studies of implants with various topographies showed that the SmoothSilk^®^ surface (Motiva^®^ Flora), with an average roughness of 4 µM, provokes the least amount of inflammation and foreign response in the rat model [45].

Hence, although tissue expanders are designated to be removed and exchanged for definite implants, their impact on the prosthesis environment must be considered beyond their period of use. Comparative studies on the clinical outcome of implant reconstruction after the use of different types of tissue expanders are scarce and they mainly focus on the evaluation of complications such as malposition, seroma formation, infections, capsular contracture, and reoperation rates [52,58,59,60]. In prior investigations, we analyzed the composition of peri-SMI connective tissue capsules only in women who received such implants for aesthetic reasons [31,61,62]. 

We hypothesize that the choice of the expander during the first operative stage may have a permanent impact on the final reconstructive result due to surface-depending tissue imprinting of the created pocket. Patients undergoing a prophylactic mastectomy followed by breast reconstruction are an ideal cohort for cellular and molecular immunological elucidation of fibrosis due to their uncompromised health status and immune system, contrary to patients with implant-based reconstruction after invasive breast cancer.

We, therefore, aimed to conduct the first intra-individual comparison and inter-individual evaluation of two different tissue expanders with varying surface topography roughness by comparing the aesthetic and patient-reported outcome as well as conducting a histopathological and radiographic evaluation of the capsule after tissue expander reconstruction with 60 µM Ra (CPX^®^4 breast, MENTOR^®^, USA; provided by Establishment Labs, Alajuela, Costa Rica) vs. 4 µM Ra (SmoothSilk^®^, Motiva Flora^®^; provided by Establishment Labs, Alajuela, Costa Rica) average surface roughness.

In immediate two-stage breast reconstruction, the expander is positioned in the submuscular/subcutaneous plane combined with a mesh pocket for fixation to the pectoralis major. Implantation of a mesh triggers a foreign-body reaction, which plays a crucial role in the incorporation of the mesh into the host tissue. By histological comparison, we opted to evaluate the potential immunoreactivity and intracapsular incorporation of Titanium-coated Polypropylene meshes. 

Our data confirm an improved surgical outcome of a reduced implant surface topography in humans–with minor capsule formation. Moreover, we demonstrate clear amplification of foreign body response by Titanium-coated implant pocket in early-stage fibrosis.

## 2. Materials and Methods

### 2.1. Study Population

This study included a total of 10 female patients with high familial risk for breast cancer, who were undergoing simultaneous prophylactic bilateral nipple-sparing mastectomy (NSME) and immediate tissue expander-based breast reconstruction. Informed consent for photo documentation, the operation, sample collection, and anonymized evaluation and publication of data was obtained in written form from all patients after confirmation of all inclusion and exclusion criteria (Table 1).

Patient demographics including age, body mass index (BMI) and breast symmetry, previous scars in the breast area, comorbidities (chronic diseases, allergies, medication), dominant hand, smoking habits, profession (manual labor/office job), and physical training habits, as well as duration, were documented. All interventions (operation, photo documentation, ultrasound, sample collection, anonymized evaluation, and publication of data) were performed, and information was obtained, with the informed written consent of the participants and in accordance with: (i) the regulations of relevant clinical research ethics committee as well as (ii) the Declaration of Helsinki and (iii) the European Union Medical Device Regulation (§40 Section 3 Medical Devices Act). The two different devices were randomized to the right or left breast, and patients were blinded. In the course of the Expander-Immunology Trial, one patient resigned, and two patients were excluded due to post-op complications. 

Therefore 7 SmoothSilk^®^ and 7 CPX^®^4 breast tissue expanders were evaluated intra- and inter-individually by patient-reported expander aesthetic and comfort outcome, surgeon-reported satisfaction with expander practicability and aesthetic outcome at informational endpoints at weeks 4, 16, and after reoperation, as well as the thickness of fibrotic capsule around expander shells.

### 2.2. Study Design

**Figure 1:**
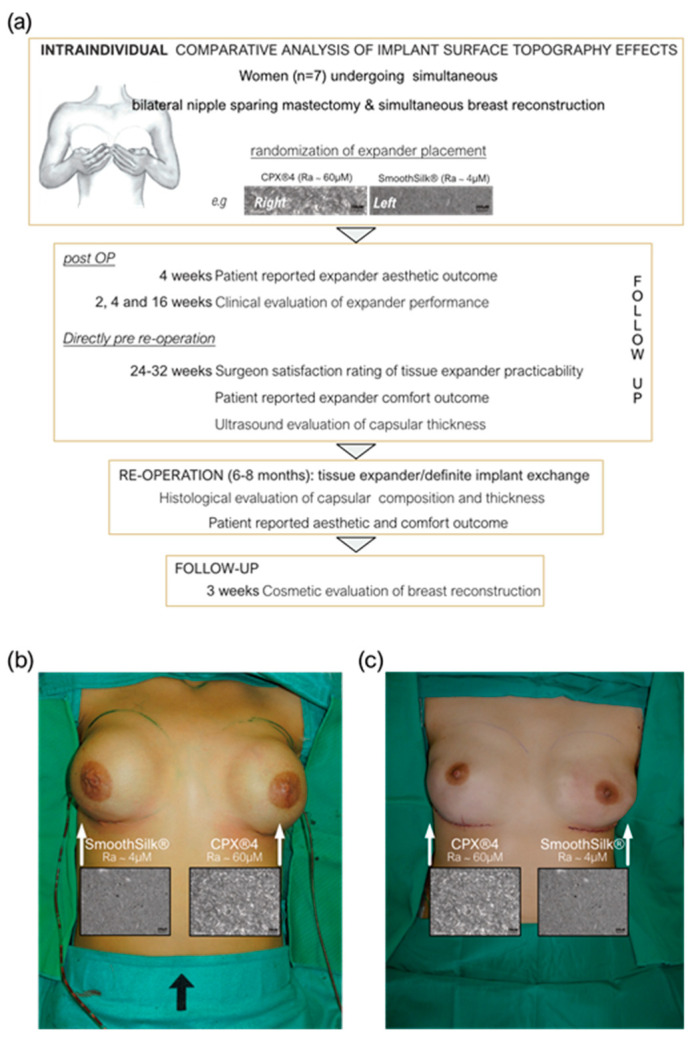
Expander immunology Trial (ClinicalTrials.gov ID: NCT05648929. (**a**) Assessment schedule pre- and after-expander implantation. (**b**,**c**) Standardized intra-operative photo documentation of an intra-individual comparative bilateral tissue expander-based breast reconstruction. Each patient received both types of expanders, the routinely used CPX^®^4 breast expanders (MENTOR^®^, USA: surface roughness: ~60 µM Ra) and the novel surface-roughness reduced SmoothSilk® breast expanders (Motiva Flora^®^, Establishment Labs, Costa Rica: surface roughness: ~4 µM Ra) randomized to the left or right breast in combination with TiLOOP^®^ pocket mesh after bilateral prophylactic NSME. (**b**) Patient 003; Right: SmoothSilk®, Left: CPX^®^4, (**c**) Patient 001; Right: CPX^®^4, Left: SmoothSilk®.

This monocentric, randomized, double-blind controlled clinical study was approved by the Institutional Ethical Committee of the Medical University Innsbruck, Austria (protocol code 1325/2019, 23 January 2020) and the Austrian Federal Office for Safety in Health Care (approval number; 13340962) and is openly accessible at ClinicalTrials.gov (ID: NCT05648929). We enrolled a total of 10 patients, who received both the routinely used expander Mentor CPX™4 and the novel Motiva SmoothSilk^®^ with reduced surface topography roughness, randomized to the left or right breast after prophylactic bilateral NSME (Figure 1). In the course of the Expander-Immunology Trial, one patient resigned, and two patients were excluded due to post-op complications. 7 patients were evaluated.

In the first operation, NSME was performed by gynecologists, subsequently, plastic surgeons performed the reconstructive part of the operation. Patients received within the first operation both tissue expanders (=inflatable silicone implants), namely SmoothSilk^®^ and CPX^®^4, to reconstruct the breasts. The expanders were positioned in the prepectoral subcutaneous plane combined with a Titan bra (TiLOOP^®^) to cover the complete frontal part of the implant. The pectoralis muscle is not in contact with the titanium mesh. Suture removal was planned 10-17 days post-op and the first extra operative expansion inflation was 28 ± 7 days post-op. Both expanders were stepwise filled during periodical follow-up care. 

Clinical examinations and questionnaire evaluation. Clinical examinations addressing any side effects of the implanted tissue expanders SmoothSilk^®^ and CPX^®^ were performed 2 weeks, 4 weeks, 5 weeks, 6 weeks, 7 weeks, 8 weeks, and 16 weeks after the initial operation. Documentation of complications (wound dehiscence, signs of inflammation, breast structure swelling, potential expander malposition) and expander symmetry as well as palpation of the breasts was conducted. Additionally, two standardized questionnaires addressing aesthetic, practicability, and comfortability outcome satisfaction of the implant were filled out 4 weeks and 6–8 months after the expander implantation. The schedule of the follow-up examinations is demonstrated in Table 2. 

Radiology. After 6–8 months, the expander was removed and a permanent silicone breast implant was placed. Directly before the expander exchange–during this hospital stay–all patients underwent an ultrasound examination preoperatively, performed by a radiologist, in order to analyze the thickness of the capsule formed around the expanders. Seroma formation, implant dislocation, and thickness of the capsule were evaluated with SIEMENS Sequoia 18L6 line (frequency: 18 MHz).

Histological processing and quantification. During reoperation, capsular tissue (3 × 3 cm) was harvested from both implants, at 2 positions, anterior contact zone with TiLOOP^®^ and posterior (TiLOOP^®^ free) contact zone with *M. pectoralis*. Samples were fixed in formalin and routinely processed at the Institute of Pathology, Neuropathology and Molecular Pathology. For histological evaluation, consecutive sections stained with Hematoxilin & Eosin (H&E) and Chromotrope-anillin-blue (CAB) were compared in parallel and capsule thickness was measured at 2 places along the implant–tissue boundary for each implant at both contact zones (n = 2 per group per implant type), for duplicate sections for lower poles or sides of the implants. 

Aesthetic outcome evaluation. The evaluation was conducted based on standardized photo documentation taken after the first (final filling of expander devices) and second stage (after exchange to final implant) of breast reconstruction. The evaluation was done by a gender-balanced panel of four independent plastic surgeons (two seniors, and two residents) using a German translation of the Breast Aesthetic Scale [63]. Seven questions of this validated tool were used to evaluate the aesthetic result for both sides in all the included seven patients after bilateral breast reconstruction. One further item about the symmetry of the breasts pertains to both sides and was therefore only answered once per patient and reconstructive stage. Every question was graded from 1 to 5, with 5 points representing the perfect aesthetic result.

Patients and plastic surgeons were double-blinded. Matching was performed intra-individually and conducted according to the implanted tissue expander. 

### 2.3. Statistical Analysis

Tissue expanders were randomized to either the left or right breast via a randomization list (provided by the Department for Medical Statistics and Health Economy, Medical University, Innsbruck). To prevent bias, the randomization list was generated prior to the first patient enrollment. Paired Student’s t-test and the non-parametric Wilcoxon test for paired samples were used for normally and non-normally distributed data sets, respectively. Variance analysis was performed for repeated measurements between the two groups. 

Statistical significance of the correlation matrix was determined by the Pearson correlation coefficient with a two-tailed confidence interval of 95%. Statistical significance of simple linear regression has been determined by comparison of slopes and intercepts with a confidence interval of 95% (inter- and intra-individual comparison; n = 7). Descriptive analysis was used to summarize patient outcome data. Statistical analyses were performed using Prism Software 9.0 (GraphPad, San Diego, California, USA). The statistical details of experiments are presented in the relevant figure legends. A *p*-value of < 0.05 was considered significant. The level for statistical significance was set at *p* ^ns^ >0.05, *p* * < 0.05, *p* ** < 0.002, *p* *** < 0.0002, and *p* **** < 0.0001, for all statistical tests. 

## 3. Results

### 3.1. Patient Characteristics

Seven healthy female patients with bilateral prophylactic mastectomy and simultaneous tissue expander-based breast reconstruction, due to high-risk hereditary predisposition and/or confirmed *Brca1+/Brca2+* gene mutation, were enrolled in the study (Figure 1a). All patients received both types of expanders, the routinely used CPX^®^4 breast expanders (MENTOR^®^, USA: surface roughness: ~60 µM Ra) and the novel surface-roughness reduced SmoothSilk® breast expanders (Motiva Flora^®^, Establishment Labs, Costa Rica: surface roughness: ~4 µM Ra) randomized to the left or right breast after bilateral prophylactic NSME (Figure 1b,c). Of note, PAT001_007 decided for a breast reconstruction and breast volume reduction. During the first operation, the prophylactic NSME was performed via a vertical incision. Subsequently, during the exchange of the expander to a definitive implant, an inverted T-reduction mastopexy was undertaken as well. Patient demographic data and device information are summarized in Table 3.

There were no significant differences in patient characteristics, mastectomy weight, implant position (prepectoral), reconstruction volume and intraoperative filling, or time point of expander exchange between the two differently textured devices (Table 4).

**Figure 2:**
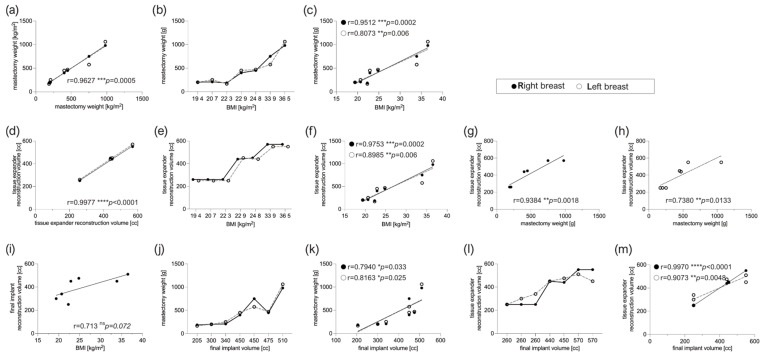
Intra-individual comparison of mastectomy weight, tissue expander reconstruction volume, and final implant volume. (**a**) Bilateral correlation of mastectomy weight obtained from left and right breast. (**b**) Mastectomy weight correlates (**c**) in a simple regression with BMI (R:w^mast^ = 44.66×BMI-698.2, L:w^mast^ = 41.38×BMI-613.4). (**d**) Bilateral correlation of tissue expander reconstruction volume obtained from left and right breast. (**e**) Tissue expander reconstruction volume correlates (**f**) in a simple regression with BMI (R:V_TE_ = 1×BMI; L:V_TE_ = 0.9585×BMI-1.352) as well as (**g**,**h**) weight of corresponding mastectomized (**g**) right and (**h**) left breast. (**i**) Final implant reconstruction volume correlation with BMI (ns). (**j**) Final reconstruction volume correlates bilaterally (**k**) in a simple regression with the weight of corresponding mastectomized breast BMI (R:V_SMI_ = 1×w^mast^; L:V_SMI_ = 0.6528×BMI-140.9) as well as (**l**,**m**) with corresponding tissue expander reconstruction volume (R:V_TE_ = 1×w^mast^; L:V_TE_ = 0.6528×BMI-140.9). Statistical significance of the correlation matrix has been determined by the Pearson correlation coefficient with a two-tailed confidence interval of 95%. Statistical significance of simple linear regression has been determined by comparison of slopes and intercepts confidence interval of 95%. The level for statistical significance was set at *p*_ns_ >0.05, * *p* < 0.05, **p < 0.002, *** *p* < 0.0002, and **** *p* < 0.0001, for all statistical tests (inter- and intra-individual comparison; n = 7).

As presented in Figure 2, the mastectomy weight of the left and right breast correlated bilaterally with high significance in all 7 subjects (Figure 2a, *** *p* = 0.005). Moreover, the mastectomy weight of both breasts correlated and increased in dependence with BMI (Figure 2b, right breast: *** *p* = 0.0002, left breast: ** *p* = 0.006). Postoperatively, the tissue expander intermediate expansion volume correlated bilaterally, but also with BMI as well as the weight of the mastectomized breast, in a simple regression, respectively. The final implant reconstruction volume correlated bilaterally with the mastectomy weight as well as with the intermediate expander volume, but not to BMI (Figure 2e,f).

### 3.2. Clinical Evaluation of Expander Performance

After NSME and expander insertion, clinical examinations were performed at 2 weeks, 4 weeks, and 16 weeks post-op. Wound dehiscence, signs of inflammation, symmetry, breast structure swelling, expander malposition, and palpation were clinically evaluated and documented (Table 5). 

**Figure 3:**
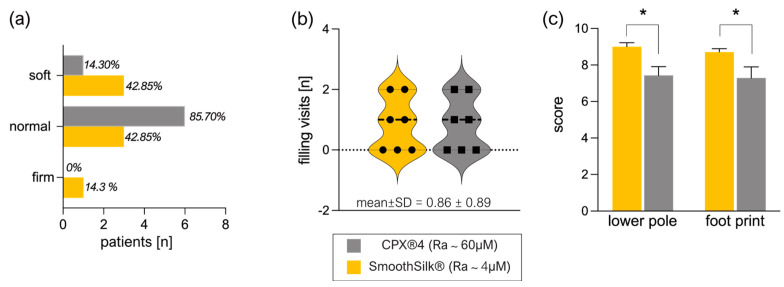
Intra–individual clinical evaluation of tissue expander performance after bilateral tissue expander-based breast reconstruction. Each patient received both types of expanders, the routinely used CPX^®^4 breast expanders (MENTOR^®^, USA: surface roughness: ~60 µM Ra) and the novel surface-roughness reduced SmoothSilk® breast expanders (Motiva Flora^®^, Establishment Labs, Costa Rica: surface roughness: ~4 µM Ra) randomized to the left or right breast after bilateral prophylactic NSME. (**a**) Evaluation of expander palpation 2 W, 4 W, and 16 W post-op. (**b**) Number of filling visits performed to full expansion of the device. (**c**) Surgeon satisfaction rating of tissue expander practicability 6–8 months post-op (lower pole: *p* * = 0.023, footprint: * *p* = 0.043; n = 7). Mean values ± SD of seven biological replicates (patients) are shown. The level for statistical significance was set at *p*_ns_ > 0.05, * *p* < 0.05, ** *p* < 0.002, *** *p* < 0.0002, and **** *p* < 0.0001, for all statistical tests (inter- and intra-individual comparison; n = 7).

None of the expanders has shown any unexpected event (e.g., seroma, infection, wound dehiscence, malposition, or rotation) during these clinical controls. Likewise, the comparison of both devices showed no significant differences in palpation (Figure 3a) and the number of expander-filling visits for the full expansion of the devices (Figure 3b). Expander practicability and aesthetic outcome evaluation of devices by the surgeon reported a high satisfaction rating (scale of 10–7: very satisfied) for the lower pole expansion and footprint of both devices (Figure 3c).

Strikingly, the standardized questionnaire revealed a significantly higher surgeon rating of satisfaction with both, the lower pole expansion (mean ± SD = 9.0 ± 0.6) and overall footprint (mean ± SD = 8.7 ± 0.5) of SmoothSilk^®^ breast expanders (Figure 3d, Table 6) compared to the rougher CPX^®^4 device (lower pole: mean ± SD = 7.4 ± 1.3: footprint: mean ± SD = 7.3 ± 1.6).

### 3.3. Patient-Reported Aesthetic and Comfort Outcome after Expander Reconstruction

The patients’ rating of overall satisfaction with the expanders (Table 6) 4 weeks after implantation indicated a mean value of 9.57 (±1.13) for the SmoothSilk^®^ breast expander and 7.86 (±1.22) for the rougher CPX^®^4 device. Although patients report generally high satisfaction with the aesthetic outcome of both devices 4 weeks post-op, patients presented more complaints about the comfortability (breast pain, breast discomfort, nipple sensitivity, and soreness) of CPX^®^4.

**Figure 4:**
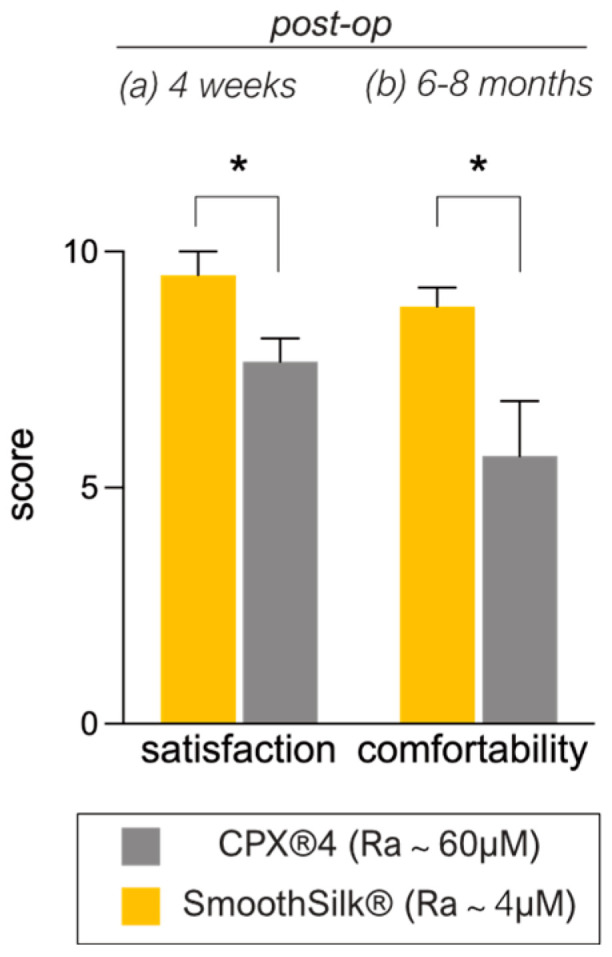
Patient (**a**) satisfaction and (**b**) comfortability rating of tissue expanders 4 weeks (**a**) and 6–8 months (**b**) post-op (satisfaction: *p* * = 0.036; comfortability: * *p* = 0.036; n = 7). Mean values ± SD of seven biological replicates (patients) are shown. The level for statistical significance was set at p_ns_ > 0.05, * *p* < 0.05, ** *p* < 0.002, *** *p* < 0.0002, and **** *p* < 0.0001, for all statistical tests. (inter- and intra-individual comparison; n = 7.

The rating of expander comfortability after total expansion time, right before the exchange to definite implants, indicated a mean value of 8.85 (±0.90) for the SmoothSilk^®^ breast expander and 6.00 (±2.77) for the rougher CPX^®^4 device. Hence, both patient-reported aesthetic and comfort outcome were significantly higher rated for the SmoothSilk^®^ breast expander (Figure 4; satisfaction after 4 weeks: *p* * = 0.036; comfortability after 6–8 M: *p* * = 0.036). 

### 3.4. Cosmetic Results

Evaluation of the aesthetic outcome was conducted by a four-member panel of plastic surgeons based on standardized photo documentation using the Breast Aesthetic Scale [63]. Intra-individual evaluation of the overall appearance showed a mean of 4.04 points for both devices and therefore did not reveal significant differences after the first stage of reconstruction (Table 7). Likewise, there was no significant difference between both expander types for all other aspects of breast aesthetics; details are given in Table 7. Evaluation of the second stage of reconstruction showed a trend towards a higher rating of the overall appearance compared to the first stage of reconstruction for both devices, but this was not found to be significant (*p* > 0.9999 in Mentor CPX^®^4 breasts and *p* = 0.7813 in SmoothSilk^®^ breasts). 

**Figure 5:**
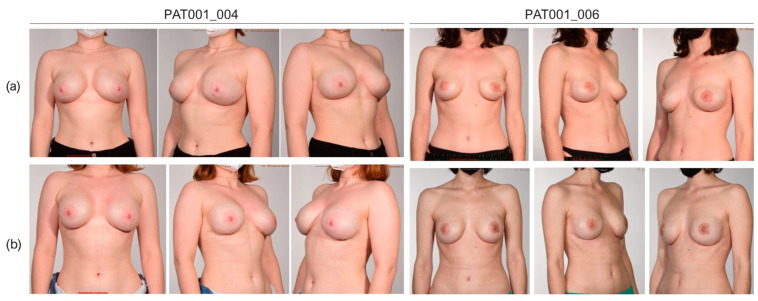
Photo documentation for cosmetic evaluation after the (**a**) first (PAT001_004 and PAT001_006; left breast: Mentor^®^CPX4, right breast: SmoothSilk^®^) and (**b**) second reconstructive stage (PAT001_004: Motiva Ergonomics SmoothSilk^®^, 450 cc, full projection, and PAT001_006: Motiva Ergonomics SmoothSilk^®^, 340 cc implants, demi).

Evaluation of both reconstructive stages showed a trend towards a better rating for the questions on symmetry, breast position, shape and contour, nipple position, and overall appearance after the second stage, as shown in Table 8. The highest increase was seen in the category shape and contour with a mean of 3.77 (±0.83) points after the expander reconstruction compared to 4.23 (±0.57) after the exchange for the final implant. Overall appearance increased from 4.04 (±0.65) points to 4.30 (±0.66) points after the second stage. The t-test did not prove the difference to be significant for all items. Results of the first and second reconstructive stages are shown in Table 8 and Figure 5.

### 3.5. Intra- and Inter-Individual Comparison of the Fibrotic Capsule Thickness Formed around the CPX^®^4 and SmoothSilk^®^ Tissue Expander

In all seven patients, the capsular tissue thickness was assessed by two different approaches, by non-invasive breast ultrasound, directly before the re-operation, and by histological analysis of peri-capsular tissue, harvested during re-operation, when expanders were exchanged for definitive implants. 

**Figure 6:**
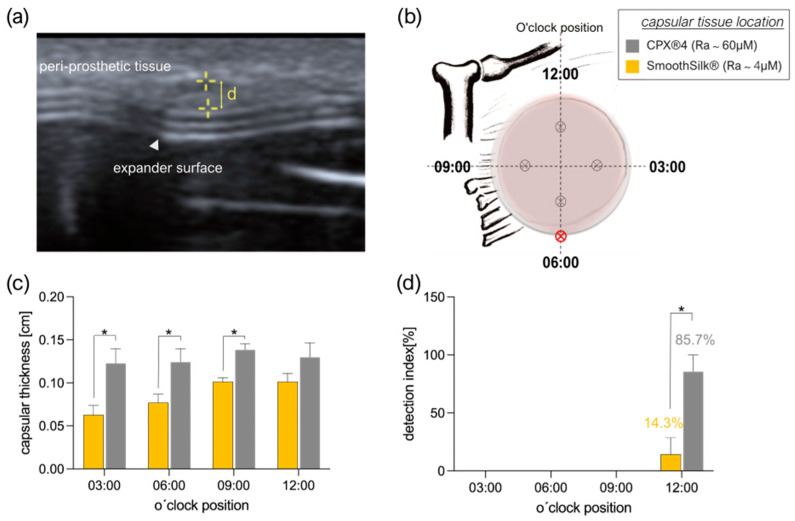
Ultrasonographic measurement of fibrotic peri-prosthetic capsule thickness of 7 patients was defined as the distance (**a**) from the silicon tissue expander surface to the outer margin of the capsule at the widest portion of the capsule and determined bilaterally at (**b**) o’clock reference positions 00:00, 03:00, 06:00, 09:00, 12:00 for the left and right breast. (**c**) Statistical significance was determined by 2-way ANOVA: O’clock position main effect: F (3,24) = 1.615, *p*^ns^ = 0.2121; expander surface roughness main effect: F (1, 24) = 39.50, **** *p*< 0.0001; position × expander roughness interaction effect: F (3,24) = 0.9667, *p*^ns^ = 0.4246. Multiple variance comparison within 4 measurement positions and post hoc Holm–Šidák *p-value* adjustment: 03:00, *** *p*_adj_ = 0.0008; 06:00, ** *p*_adj_ = 0.0088; 00:09, * *p*_adj_ = 0.0490; 12:00, ^ns^ *p*_adj_ = 0.1807. (**d**) Detection of periprosthetic fluid deposits bilaterally at all 4 of the o´clock positions. Statistical significance was determined by 2-way ANOVA: O’clock position main effect: F (3,48) = 24.50, **** *p*< 0.0001; expander surface roughness main effect: F (1,48) = 12.50, *** *p* = 0.0009; position × expander roughness interaction effect: F (3,48) = 12.50, ****p< 0.0001. Multiple variance comparison within 4 measurement positions and post hoc Holm–Šidák *p-value* adjustment: 03:00, ^ns^ *p*_adj_ < 0.9999; 06:00, ^ns^ *p*_adj_ < 0.9999; 09:00, ^ns^ *p*_adj_ < 0.9999; 12:00, **** *p*_adj_ < 0.0001. Mean ± SEM of 7 patients in 7 independent experiments is shown. The level for statistical significance was set at p_ns_ > 0.05, * *p* < 0.05, ** *p* < 0.002, *** *p* < 0.0002, and **** *p* < 0.0001, for all statistical tests (inter- and intra-individual comparison; n = 7).

Directly before the expander was exchanged, one single experienced radiologist (expert for breast ultrasound) determined capsular thickness, liquid accumulation, and signs of expander dislocation in a standardized o’clock position measurement (Figure 6a). An illustrative case of measurement position is shown in Figure 6b. Overall, ultrasound examination confirmed that the fibrotic capsule thickness on the rougher CPX^®^4 tissue expander was significantly thicker than the capsule formed on SmoothSilk^®^ expanders (Figure 6c; **** *p* < 0.0001) at 3 of the 4 positions assessed; however, this was not found at 12:00 o’clock. Additionally, we detected exclusively in the 12 o’clock position, in the upper central part of the breast, periprosthetic fluid deposits around both expanders next to the port, with a significant increase around the rougher CPX^®^4 device (Figure 6d). However, expander identification, due to technical reasons (SmoothSilk^®^; integrated port), was not possible for the radiologist during the ultrasound assessment. Ultrasound assessment documentation for every patient will be provided upon request.

### 3.6. Titanium Debris from TiLoop^®^ Bra Increases Histopathological Changes of the Capsule

**Figure 7:**
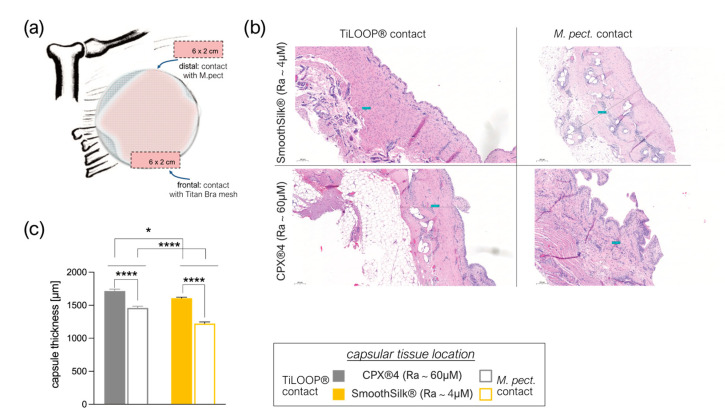
The thickness of the capsule formed around tissue expanders 6-8 months after bilateral tissue expander-based breast reconstruction. (**a**) Sample collection scheme for histological analysis of capsular thickness. (**b**) Hematoxylin and eosin stain of post-operative collected capsular tissue. (**c**) The thickness of the capsule of 7 patients was determined bilaterally in 2 biological replicates and 2 measurements per trial. Mean ± SEM of 7 patients in 7 independent experiments is shown. Statistical significance was determined by 2-way ANOVA: Capsular harvest position (contact zone) main effect: F (1, 16) = 176.6, **** *p* < 0.0001; expander surface roughness main effect: F (1, 16) = 50.63, **** *p* < 0.0001; harvest position × expander roughness interaction effect: F (1, 16) = 7.026, * *p* = 0.0174. The level for statistical significance was set at *p*_ns_ >0.05, * *p* < 0.05, ** *p* < 0.002, *** *p* < 0.0002, and **** *p*< 0.0001, for all statistical tests (inter- and intra-individual comparison; n = 7).

We studied 20 excised capsules in five patients, with samples collected (Figure 7a) anterior at the TiLOOP^®^/expander covered zone, and posterior at the expander/*M. pectoralis* contact zone in both breasts, around both tissue expanders. Comparison of fibrotic capsule thickness (Figure 7b) revealed not only a significantly thinner capsule around SmoothSilk^®^ expanders with reduced surface roughness (Ra~4 µM (Figure 7c; frontal contact zone: * *p* = 0.0361, distal zone *** *p* < 0.0001)), but also a biologic effect of the Titan containing TiLOOP^®^ pocket Bra, used for expander pocket fixation. The capsule was significantly thicker at the frontal TiLOOP^®^/expander contact zone around both expanders compared to the titan-free location at the distal *M. pectoralis*/expander contact zone (Figure 6c, CPX^®^4: **** *p* < 0.0001, SmoothSilk^®^: **** *p* < 0.0001).

## 4. Discussion

Despite clear evidence of superior long-term satisfaction after autologous breast reconstruction, the US, as well as Europe have experienced a clear shift towards implant-based breast reconstruction. 

Silicone is the most widely used implant material in routine medical practice, despite side effects, such as the formation of hypertrophic fibrotic peri-implant capsules causing pain, local tissue damage, and impairment of implant function [64,65,66,67]. Surgical implantation of a biomaterial, no matter how noninvasive, causes injury that can initiate the fibrotic response [68,69]. Any injury leads to inflammation, matrix formation, and matrix rearrangement [31]. If the infection is associated with the biomaterial, the degree of fibrosis will increase dramatically [69]. 

Biomaterial surface chemistry, mechanical properties, and topography have been shown to influence the ultimate immune response [68,70]. However, depending on the topography of these surfaces, varying degrees of capsular fibrosis are reported [46,50,71] as well as the finding that implants with an average roughness of 4 μm provoke the least amount of inflammation and foreign body response [45]. Based on these histo-morphological analyses, a comparative evaluation of surface texture effect on implant encapsulation in patients and a controlled clinical setting is definitely required.

In this study, we intra- and inter-individually compared two commercial silicone breast tissue expanders with varied surface topographies: SmoothSilk^®^ (Motiva; Ra ~ 4 µM) and CPX^®^4 (Mentor Ra ~ 60 µM) in a prophylactic setting. All patients received both devices, randomized to the left or right breast to define the consequences of SMI surface topography on specific outcomes: postoperative complications, implant rippling or malposition, and the aesthetic outcome. 

In the course of intra-individual device performance comparison, no matching of patient groups was done. As both devices were inserted into every subject, no significant differences in patient characteristics, mastectomy weight, implant position (prepectoral), reconstruction volume and intraoperative filling or time point of expander exchange within comparative groups (SmoothSilk^®^ vs. CPX^®^4) could be determined. Weight of both mastectomized breasts correlated not only bilaterally (right vs. left), but also in a simple regression with BMI, intermediate expansion volume of expanders, and final reconstruction volume of implants. The intermediate expansion volume and final implant reconstruction volume correlated bilaterally with each other and with BMI. 

Concerning the BMI, all enrolled subjects could be discriminated as either normal weight (18.5–24.9 kg/m^2^; n = 5) or obese (BMI > 30; n = 2). A BMI > 30 correspondingly also increased the weight of both breasts to w^mast^ > 500 g. While smaller breasts (w^mast^ < 500 g) have been reconstructed according to their initial weight, mastectomized breasts with a weight higher than 500 g were reconstructed with T form reduction, though the final implant reconstruction volume did not correlate regressively with the BMI.

For women undergoing risk-reducing mastectomy to prevent breast cancer, reconstruction can be challenging in those with larger breasts. Large implants can increase the risk of complication rates, implant rippling, and capsular contracture. Additional surgery may be needed to correct an over-endowment in implant size [72]. To improve long-term aesthetic outcome and correspond to patients’ will, we reduced breast size during reconstructive surgery. 

Shell surface roughness variation did not affect post-operative complications evaluated in follow-up clinical examinations 2, 4, and 16 weeks post-operatively. There was no difference in filling attempts, procedure, or volume (intra-individually). Moreover, wound status and symmetry were comparable for both devices. Of note, in a recently published short-term prospective study, SmoothSilk^®^ has shown a low rate of overall complications to date [73].

Crucially, in our study, clinical and visual evaluation of tissue expander performance and practicability, by the surgeon, revealed significant benefits of the SmoothSilk^®^ device. Benefits included improved palpation, as the device was perceived as softer compared to CPX^®^4, and lower pole expansion creating an ideal footprint of the breast. 

Importantly, patients shared the surgeon’s practicability rating, very early after reconstruction (4W) and perceived the SmoothSilk^®^ expander as significantly more comfortable compared to CPX^®^4. The correlation between surgeon and patient satisfaction reports is worth mentioning, as Wu and colleagues recently showed the striking effect of socioemotional determinants on patient and observer perception discrepancies in the assessment of aesthetic outcomes [74]. This might be due to technical differences of the expanders, as the filling port of CPX^®^4 is supported with magnetic metal inclusions, making it stiff and possibly irritating that position internally.

Aesthetic evaluation of both devices used for the first stage of reconstruction did not reveal significant differences. Likewise, cosmetic results were comparable after the second stage of reconstruction. Nevertheless, the impact of the type of expander on long-term results must be considered, since the formation of the capsule around the device takes place during the expansion period. Therefore, a permanent tissue footprint may influence the reconstructive results beyond this period. This may be viewed in the context of our radiological findings, where periprosthetic fluid and fibrotic capsule thickness were significantly increased around Mentor^®^CPX4 expanders. Furthermore, this is also strongly confirmed with the conducted histological analysis where the capsule tissue around SmoothSilk^®^ expander devices was found to be significantly thinner. We hypothesize that these findings may give a hint about the inflammatory processes in the surrounding tissue and therefore may be linked to long-term complications such as capsular contracture or late seroma. In this study, mean follow-up was 36 days after device exchange; therefore, no conclusions about long-term results can be drawn. This represents a limitation of our study, but we aim to conduct a sequel study evaluating the long-term results of our patients. 

Our data suggest a trend towards better cosmetic results after the second stage of reconstruction, although this was not significant. Buck et al. revealed a significant improvement in cosmesis scores after the evaluation of different stages of reconstruction in their study [75]. We think that information about changes throughout the reconstructive period and the importance of completion must be part of the initial patient counseling to keep up patience for the final result and avoid rash frustration in patients. In our experience, performance of the expander device is linked to the filling volume. We have observed that exhaustion of the expansion volume or even slight overfilling might lead to deformations, such as the prominence of the upper pole or even the rectangular shape of the expander (Figure 5a). Therefore, we prefer to stay under the maximum filling, but evaluation of these observations must be done in a further study. 

Breast tissue expanders with magnetic ports, like CPX^®^4, are magnetic resonance imaging (MRI) unsafe; therefore, non-invasive radiology is applied for diagnosing implant complications, implant displacement, or rotation. Ultrasound evaluation revealed significantly reduced capsules around SmoothSilk^®^ expanders and a highly significant occurrence of seroma formation in the upper pole of the CPX^®^ expander. The importance of this finding cannot be emphasized too much, as we suspect the stiffness of the magnetic filling port in CPX^®^4 is the cause of irritation and tissue disruption at that position internally. As already mentioned, the SmoothSilk^®^ device not only differs in surface topography but also in the nonmagnetic filling port with an incorporated radio frequency identification device to offer MRI scanning during the tissue expansion process [76]. Additionally, the SmoothSilk^®^ expander might be identifiable by the radiologist during ultrasound assessment through three fixation points, thus permitting precise identification of the position through non-invasive, non-magnetic evaluation of displacement and rotation. Despite the clear benefits in terms of traceability, the absence of a stiff metallic region on the expander surface clearly reduces the foreign body response in the form of capsular fibrosis around the SmoothSilk^®^ device.

A variation in histomorphology was observed between samples. In general, the capsule region adjacent to the implant lacked vascularization, although vascularization throughout the entire capsule was evident in samples. After 6-8 months we found uncontracted capsules formed around both tissue expanders, composed of thin, loosely arranged, multidirectional, string-like fibers. Morphology consistent with synovial metaplasia was observed in some samples and was characterized by a layer of synovial-like cells arranged in a palisaded manner at the capsule-implant interface. Analysis of capsule thickness confirmed pre-operative ultrasound evaluation: tissue encapsulating SmoothSilk^®^ expanders is significantly thinner in comparison with CPX^®^4.

SMI design has been altered and improved over the years, including changes to the cohesiveness of the silicone filler gel and texturing of the shell. However, the basic device design remains a silicone elastomer shell surrounding a viscous silicone gel, and biocompatibility, as well as safety, have been a source of long-standing controversy. It is not only important to consider the effects of the elastomer shell but also of the filler gel.

Our findings of intracapsular silicone droplet inclusion substantiate previous findings of breast implant bleeding and debris-associated pathogenicity [77,78,79] over time; SMI rupture can appear by tears that occur in the implant shell, which often remain undetected. Virtually all SMIs will bleed components from the filler gel; moreover, there is emerging evidence of severe biologic reactivity to breast implant particulate shedding [77]. Both are associated with biological profibrotic hypersensitivity reactions and compromise the implant’s long-term clinical performance.

Moreover, our data clearly reveal a TiLOOP^®^ Bra Pocket conditioned increase in capsule thickness generally. Titanium-coated polypropylene meshes are a helpful device in breast surgery, where the prepectoral use of tissue bridging materials is indicated to fix the Pectoralis major and secure the position of the SMI. However, these meshes have shown a variety of complication rates [80,81]. Recently, Titan mesh incorporation in breast soft tissue after reconstruction was verified [82].

Up to this point, our clinical, radiological and histological impression is that the SmoothSilk^®^ tissue expander with reduced surface roughness reduces fibrotic capsule formation and accumulation of periprosthetic fluid. Clinically, the improved biocompatibility is reflected in an improved aesthetic, practicability, and patient comfort outcome.

From a clinical point of view, capsular contracture, the most prominent complication associated with SMI, is defined by its symptoms, namely palpable hardness, visible deformities, or pain, of which the latter is an absolute indication for surgical intervention.

Therefore, evaluation of capsule thickness and its effects on implant performance (position, rotation) and wound healing as well as patients´ well-being is inevitable for the detection of clinical symptoms and therapeutic consequences. To sum up, reducing SMI shell surface roughness to Ra 4 µM can achieve an improved clinical and aesthetic outcome in breast reconstruction.

In general, SMI is designed to be nontoxic and non-immunogenic while keeping an active role in host response, which–we know–plays an active role in fibrogenesis. There is evidence to suggest that biomechanical parameters, such as tensile strength, elasticity, and permeability, of both tested devices should be carefully evaluated over the long term, as permeable gel leakage and particulate shedding through the outer shell cause serious health damage. By the same token, the fixation of breast implants with titanised, lightweight polypropylene meshes needs to be re-evaluated in terms of biocompatibility.

## 5. Conclusions

Investigated in patients for the first time, our data confirm an improved immune biocompatibility of a reduced implant surface topography in humans, with minor capsule formation. This may lead in the longer term to lower revision rates and a better aesthetic outcome accompanied by higher patient satisfaction.

## Figures and Tables

**Table 1:** Inclusion and exclusion criteria for the Expander-Immunology trial.

Inclusion Criteria		Exclusion Criteria
Female sex	**1**	Sever coagulation disorder, representing a potential contraindication for the elective surgery
Age > 18 years	**2**	Rheumatic disease accompanied by oblkigatory intake of immunomodulating therapeutic agents
High-risk family history for breast and/or ovarian cancer and/or BRCA1/2 gene mutation carrier	**3**	Severe renal functional disorder: renal insufficiency status IV or V (estimated glomerulary filtration rate (GFR) < 30 mL/min)
Planned bilateral mastectomy with simultaneous breast reconstruction	**4**	Active hematological or oncological disease
Signed Informed consent form.	**5**	HIV-Infection
	**6**	Hepatitis-Infection
	**7**	Pregnancy or breast-feeding
	**8**	Intake of anti-inflammatory drugs
	**9**	Carrier of silicone implants (e.g., gastric banding, mammary implants)

**Table 2:** Satisfaction questionnaire. Surgeon and patient scale of device aesthetic, practicability, and comfortability outcome, as well as satisfaction after expander-based breast reconstruction with SmoothSilk^®^ and CPX^®^4.

Surgeon Scale	Time Point Post Op	Numeric Scale
How satisfied are you with the lower pole expansion of the expander?	6–8 M	not satisfied: 0 to 3 satisfied 4 to 7 very satisfied: 8 to 10
How satisfied are you with the footprint created by the expander?	6–8 M	
**Patient Scale**	**Time Point Post Op**	
How satisfied are you with the expander?	4 W	not satisfied: 0 to 3 satisfied 4 to 7 very satisfied: 8 to 10
Please indicate how comfortable the expander was?	6–8 M	not comfortable: 0 to 3 comfortable 4 to 7 very comfortable: 8 to 10

**Table 3:** Summary of patient demographic characteristics and device information.

*IMPLANTATION SITE*	Left: SmoothSilk^®^; Right: Mentor CPX4			Left: Mentor CPX4; Right: SmoothSilk^®^			
	PAT 001_001	PAT 001_002	PAT 001_007	PAT 001_003	PAT 001_004	PAT 001_005	PAT 001_006
Vital Parameters							
age (y)	34	41	30	31	26	60	33
weight (kg)	86,3	54	108	54	58	105	57
size (cm)	186,5	155,5	172	167	159	176	166
BMI	24.8	22.3	36.5	19.4	22.9	33.9	20.7
body surface area	2.12	1.52	2.9	1.6	1.59	2.2	2.7
Status of natural breast							
asymmetry	no	no	no	no	no	no	no
scars	no	no	no	no	no	no	no
diseases	no	no	no	no	no	no	no
active smoker	yes	no	no	no	no	no	no
allergies	no	no	no	no	no	no	no
Chronic diseases							
diabetes	no	yes	no	no	no	no	no
Other							
job	manual job	office job	office job	office job	office job	manual job	office job
physical training (h/week)	>2	>2	0.5–2	>2	>2	0.5–2	0.5–2
dominant hand	right	right	right	right	right	right	right
1st operation: tissue expander implantation							
Bilateral prophylactic NSME resection weight [g]							
right breast	449	187	980	200	400	750	208
left breast	471	167	1060	200	450	575	252
Prepectoral reconstruction volume [cc]							
Motiva Flora^®^ SmoothSilk^®^	450	260	570	260	440	570	260
Mentor CPX4	440	250	550	250	450	550	250
Intrapoerative filling (both devices)	250	150	550	150	300	500	150

KOF is a german definition for body surface area.

**Table 4:** Intra-individual statistical comparison of analytical groups.

	SmoothSilkⓇ		Mentor CPX4		
Surface Roughness	Ra ~ 4 µM		Ra ~ 60 µM		
	Mean	(±std)	Mean	(±std)	*p* Value
age (y)	35.2	11.4	35.2	11.4	intraindividual comparison >0.9999
weight (kg)	71.4	24.5	71.4	24.5	
size (cm)	168.6	10.5	168.6	10.5	
BMI	25.1	6.7	25.1	6.7	
Bilateral prophylactic NSME resection weight [g]					
left breast	434.9	404.0	436.9	454.0	0.993196
right breast	334.2	257.5	337.9	174.4	0.975407
Prepectoral reconstruction volume [cc]					
left breast	405.5	156.3	392.6	151.8	0.877595
right breast	360.8	151.1	352.7	150.0	0.920737
intaoperative filling [mL]	254.7	169.4	254.7	169.4	intraindividual comparison >0.9999
exchange time point [d]	204.8	25.8	204.8	25.8	

**Table 5:** Clinical evaluation of tissue expander performance 2 W, 4W, and 16 W post-device implantation.

		SmoothSilk^®^		Mentor CPX4		
	Surface Roughness	Ra ~ 4 µM		Ra ~ 60 µM		
	Time Point Post Op	Mean	(±std)	Mean	(±std)	*p* Value
Wound dehiscence	2 W, 4 W, 16 W	no		no		>0.9999
Signs of inflammation	2 W, 4 W, 16 W	no		no		>0.9999
symmetry	2 W, 4 W, 16 W	yes		yes		>0.9999
Breast structure swelling	2 W, 4 W, 16 W	no		no		>0.9999
Expander malposition	2 W, 4 W, 16 W	no		no		>0.9999
filling visits	6–8 M	0.86	0.90	0.86	0.90	>0.9999
Palpation						
firm	2 W, 4 W, 16 W	1 (14.30%)		0 (0%)		
normal	2 W, 4W, 16 W	3 (42.85%)		6 (85.70%)		
soft	2 W, 4 W, 16 W	3 (42.85%)		1 (14.30%)		

**Table 6:** Satisfaction questionnaire. Surgeon and patient scale of device aesthetic, practicability, and comfortability outcome satisfaction.

		SmoothSilk^®^		Mentor CPX4		
	Surface Roughness	Ra ~ 4 µM		Ra ~ 60 µM		
Surgeon scale	time point post op	Mean	(±std)	Mean	(±std)	*p* value
How satisfied are you with the lower pole expansion of the expander?	6–8 M	9.00	0.63	7.33	1.37	0.021872 *
How satisfied are yopu with the footprint created by the expander?	6–8 M	8.67	0.52	7.17	1.72	0.068264
**Patient scale**	**time point post op**	**Mean**	**(±std)**	**Mean**	**(±std)**	***p*** **value**
How satisfied are you with the expander?	4W	9.50	1.22	7.67	1.21	0.026164 *
Please indicate how comfortable the expander was?	6-8M	8.83	0.98	5.67	2.88	0.028731 *
**numeric scale**	very satisfied: 10 to 7		satisfied 7 to 4		not satisfied: 3 to 0	
	very comfortable: 10 to 7		comfortable 7 to 4		not comfortable: 3 to 0	

The level for statistical significance was set at *p*_ns_ >0.05, * *p* < 0.05, ** *p* < 0.002, *** *p* < 0.0002, and ****p < 0.0001, for all statistical tests (inter- and intra-individual comparison; n = 7).

**Table 7:** Aesthetic evaluation of Mentor CPX^®^4 and SmoothSilk^®^ expander devices after the first and second stages of reconstruction.

	First Stage of Reconstruction					Second Stage of Reconstruction				
Expander Type	Mentor CPX4 *		SmoothSilk^®^ *			Mentor CPX4 *		SmoothSilk^®^ *		
Surface Roughness	Ra ~ 60 µM		Ra ~ 4 µM			Ra ~ 60 µM		Ra ~ 4 µM		
Questions	Mean	(±std)	Mean	(±std)	*p*-Value	Mean	(std)	Mean	(std)	*p*-Value
**Breast**										
Breast position	4.04	(0.57)	4.07	(0.59)	0.9173	4.25	(0.50)	4.25	(0.58)	>0.999
Inframammary fold	4.50	(0.44)	4.21	(0.56)	0.3454	4.38	(0.49)	4.50	(0.48)	0.6933
Volume	4.36	(0.35)	4.36	(0.35)	>0.999	4.38	(0.66)	4.29	(0.68)	0.8482
Shape and contour	3.79	(0.85)	3.75	(0.81)	0.9419	4.29	(0.53)	4.17	(0.61)	0.7355
**Scar**										
Appearance	4.54	(0.47)	4.46	(0.59)	0.8205	4.50	(0.52)	4.54	(0.44)	0.8943
**Nipple-Areola Complex**										
Nipple position	4.29	(0.51)	4.29	(0.63)	>0.999	4.58	(0.47)	4.29	(0.73)	0.4693

* Device used during 1st stage of reconstruction.

**Table 8:** Aesthetic evaluation of cosmetic results after the first and the second stage of reconstruction.

	First Stage		Second Stage		
Questions	Mean	(±std)	Mean	(±std)	*p*-Value
**Breast**					
Symmetry	4.29	(0.54)	4.42	(0.49)	0.6850
Breast position	4.05	(0.58)	4.25	(0.54)	0.4035
Inframammary fold	4.36	(0.52)	4.44	(0.49)	0.7031
Volume	4.36	(0.35)	4.33	(0.67)	0.9125
Shape and contour	3.77	(0.83)	4.23	(0.57)	0.1326
**Scar**					
Appearance	4.50	(0.53)	4.52	(0.48)	0.9216
**Nipple-Areola Complex**					
Nipple position	4.29	(0.57)	4.44	(0.63)	0.5428
**Overall Appearance**	4.04	(0.65)	4.31	(0.71)	0.3271

## Data Availability

The data presented in this study are available on request from the corresponding author.
